# A Prospective Audit Investigating How the Anticholinergic Effect of Medications on Cognition Is Assessed and Reduced in Patients Admitted to an Older Adult Ward in a Psychiatric Hospital

**DOI:** 10.1192/bjo.2025.10630

**Published:** 2025-06-20

**Authors:** Dinushi Marambe, Prami Rai

**Affiliations:** Kent and Medway NHS and Social Care Partnership Trust, Canterbury, United Kingdom

## Abstract

**Aims:** Older adults often have multiple comorbidities associated with an increased risk of anticholinergic effects. Mental health medications increase this risk, contributing to cognitive decline, dementia, memory loss and confusion. Evidence suggests stopping these medications can reduce cognitive deterioration and progression of dementia. MediChec is an online tool that calculates an Anticholinergic Effect on Cognition (AEC) score. An AEC score above 2 and a total AEC score above 3 require a medication review for potential adjustment or deprescribing. This audit aims to determine whether the AEC scores were recorded at admission, during admission, and at discharge. Additionally, it assesses if further actions were taken to deprescribe unsuitable anticholinergics, resulting in a lower AEC score before discharge.

**Methods:** Data was collected using RIO (clinical system) and eMeds (prescribing system) for inpatients admitted to the older adult ward, from 1 August 2024 to 9 November 2024. Patients’ past and current medications, including their AEC scores documented in the Notes section, were reviewed on eMeds. RIO notes were used to determine whether side effects were reconsidered during ward reviews with the medical team.

**Results:** 24 inpatients were identified with AEC score assessments documented for 22 inpatients. 22.7% (n=5) of these assessments were performed within the first 7 days of admission. The AEC scores recorded were documented solely by the lead pharmacist.

Antidepressant use was noted in 45.5% (n=10) of those 22 patients. Among these, 90% (n=9) were prescribed medication with an AEC score of 1, while the remaining had a score of 2. Similarly, 50% (n=11) of the patients were on antipsychotic medication. On the AEC score assessment, 27% (n=3) of these medications scored 1, 64% (n=7) scored 2, and 9% (n=1) scored 3.

For the above patients whose AEC scores indicated a review, 0 patients went on to receive a documented medication review and follow-up AEC score.

**Conclusion:** Increasing awareness and understanding of the anticholinergic effect on cognition can help provide better patient care. This can be achieved by sending a poster about the importance of reviewing medication for higher AEC scores to the pharmacy team and the ward doctors. Likewise, implementing the AEC score in the ward-round template and recording any changes to the medication on RIO and eMeds. By utilising the AEC score to guide medication reviews and deprescribing regimes, the cognitive burden of anticholinergic medications may be significantly reduced and ultimately promote improved outcomes.

